# An Explainable Vision Transformer Model Based White Blood Cells Classification and Localization

**DOI:** 10.3390/diagnostics13142459

**Published:** 2023-07-24

**Authors:** Oguzhan Katar, Ozal Yildirim

**Affiliations:** 1 Department of Software Engineering, Firat University, Elazig 23119, Turkey; okatar@firat.edu.tr; 2 Department of Artificial Intelligence and Data Engineering, Firat University, Elazig 23119, Turkey

**Keywords:** vision transformers, white blood cells, explainable AI models, deep learning, Score-CAM

## Abstract

White blood cells (WBCs) are crucial components of the immune system that play a vital role in defending the body against infections and diseases. The identification of WBCs subtypes is useful in the detection of various diseases, such as infections, leukemia, and other hematological malignancies. The manual screening of blood films is time-consuming and subjective, leading to inconsistencies and errors. Convolutional neural networks (CNN)-based models can automate such classification processes, but are incapable of capturing long-range dependencies and global context. This paper proposes an explainable Vision Transformer (ViT) model for automatic WBCs detection from blood films. The proposed model uses a self-attention mechanism to extract features from input images. Our proposed model was trained and validated on a public dataset of 16,633 samples containing five different types of WBCs. As a result of experiments on the classification of five different types of WBCs, our model achieved an accuracy of 99.40%. Moreover, the model’s examination of misclassified test samples revealed a correlation between incorrect predictions and the presence or absence of granules in the cell samples. To validate this observation, we divided the dataset into two classes, Granulocytes and Agranulocytes, and conducted a secondary training process. The resulting ViT model, trained for binary classification, achieved impressive performance metrics during the test phase, including an accuracy of 99.70%, recall of 99.54%, precision of 99.32%, and F-1 score of 99.43%. To ensure the reliability of the ViT model’s, we employed the Score-CAM algorithm to visualize the pixel areas on which the model focuses during its predictions. Our proposed method is suitable for clinical use due to its explainable structure as well as its superior performance compared to similar studies in the literature. The classification and localization of WBCs with this model can facilitate the detection and reporting process for the pathologist.

## 1. Introduction

White blood cells (WBCs), also known as leukocytes, play a vital role in the body’s immune response [1]. They are produced in the bone marrow and are an essential component of the body’s defense system against infection and disease. WBCs are classified as either granulocytes, which possess granules in their cytoplasm, or agranulocytes, which lack granules [2]. Granulocytes include neutrophils, eosinophils, and basophils. Agranulocytes include lymphocytes and monocytes. Neutrophils, the most common type of WBCs, are the first to arrive at the site of an infection and are responsible for engulfing and destroying bacteria and other foreign particles [3]. Lymphocytes include T and B cells, which are responsible for cell-mediated and antibody-mediated immunity, respectively [4]. T cells help to identify and attack infected or cancerous cells, while B cells produce antibodies that can neutralize pathogens. Monocytes mature into macrophages, which consume and destroy microorganisms and debris [5]. Eosinophils play a role in the body’s response to parasitic infections and allergies [6]. Basophils release histamine and other inflammatory chemicals in response to allergens and other stimuli [7].

WBCs number can increase in response to infection, inflammation, or other stimuli. An abnormal increase in WBCs count is called leukocytosis, while a decrease is called leukopenia [8]. Abnormalities in WBCs counts can indicate a variety of medical conditions, including infections, cancers, and immune system disorders. A complete blood count (CBC) test, which isolates WBCs from a blood sample and studies their number and appearance under a microscope, is commonly used as part of a routine medical check-up [9].

The utilization of artificial intelligence-based systems to automatically classify WBCs in a CBC test can provide several benefits. Firstly, it can enhance the accuracy and consistency of the results by removing the subjective nature of manual classification. Manual classification of WBCs is a complex and time-consuming task that requires a high level of expertise and experience [10]. However, with AI-based systems, the process can be automated, and the results can be more consistent, as the system does not get tired or make mistakes due to human error. Secondly, it can also increase the efficiency of the process by reducing the time required for manual classification. This can be especially beneficial in high-volume settings, such as in hospital laboratories, where a large number of CBC tests are performed daily. Automated classification can also help to reduce the workload of laboratory staff, allowing them to focus on other tasks. Furthermore, AI-based systems can also provide additional information that may not be visible to the human eye, such as detecting rare or abnormal cells, which can assist in the diagnosis of certain blood disorders.

In recent years, there has been a growing interest in using machine learning and artificial intelligence to automate the analysis of WBCs. Deep learning algorithms have been employed to develop automated systems that can identify and segment WBCs in digital images of blood samples, providing a faster and more accurate alternative to manual analysis [11]. To perform WBCs classification using deep learning, a dataset of labeled images is first employed to train a neural network model. The model is subsequently able to make predictions on new images, accurately identifying and classifying various types of WBCs. This approach has demonstrated promising results, with some studies showing the ability to achieve high levels of accuracy and precision in WBCs classification [12,13,14].

A number of studies have explored the utilization of deep learning for WBCs classification, employing techniques such as convolutional neural networks (CNNs) and recurrent neural networks (RNNs) to classify various types of WBCs. Cheuque et al. [15] proposed a two-stage hybrid multi-level scheme for efficiently classifying four groups of WBCs (lymphocytes, monocytes, segmented neutrophils, and eosinophils) using a combination of a Faster R-CNN network and parallel CNNs with the MobileNet structure. The proposed model achieved a performance metric of approximately 98.4% in terms of accuracy, recall, precision, and F-1 score. Sharma et al. [16] proposed a deep learning model, specifically the DenseNet121 model, for classifying various types of WBCs in blood cell images. They utilized preprocessing techniques, such as normalization and data augmentation, to optimize the model. The model was evaluated using a dataset from Kaggle containing 12,444 images of various types of WBCs. The results indicated that the model achieved an accuracy of 98.84%, precision of 99.33%, sensitivity of 98.85%, and specificity of 99.61%. Jung et al. [17] proposed a CNN-based method, referred to as W-Net, for WBCs classification, which was evaluated on a large-scale dataset of real images of the five types of WBCs. The proposed method, W-Net, achieved an average accuracy of 97% and demonstrated superior performance compared to other CNN and RNN-based model architectures. The authors also proposed the utilization of Generative Adversarial Networks (GANs) to generate synthetic WBCs images for educational and research purposes. Rustam et al. [18] proposed a hybrid feature set that combines texture and RGB features from microscopic images for classifying various types of WBCs in blood cell images. They utilized a synthetic minority oversampling technique-based resampling to mitigate the influence of imbalanced datasets, which is a common problem in existing studies. The authors also adopted machine and deep learning models for performance comparison using the original dataset, augmented dataset, and oversampled dataset to analyze the performances of the models. The results suggest that a hybrid feature set of both texture and RGB features from microscopic images, yields a high accuracy rate of 97.00% with random forest. Chola et al. [19] proposed a deep learning framework, referred to as BCNet, for the identification of various types of blood cells in an eight-class identification scenario. The proposed BCNet framework is based on transfer learning with a CNN. The dependability and viability of BCNet were established through exhaustive experiments consisting of five-fold cross-validation tests. The performance of BCNet was compared with state-of-the-art deep learning models such as DenseNet, ResNet, Inception, and MobileNet. The BCNet framework achieved the highest performance with the RMSprop optimizer, with 98.51% accuracy and 96.24% F-1 score. Ahmad et al. [20] proposed a hybrid methodology for the classification of WBCs. They utilized a synthetic dataset comprising five distinct types of WBCs, with each class consisting of 1000 samples. The researchers conducted feature extraction on the dataset samples by employing pre-trained DarkNet-53 and DenseNet-201 models. Subsequently, they performed feature selection using the entropy-controlled marine predators algorithm (ECMPA). The support vector machine (SVM) classifier achieved an accuracy of over 99% when applied to the selected features. Bairaboina et al. [21] developed a deep learning model aimed at classifying WBCs, based on images of peripheral blood smears. The researchers employed a GhostNet-based deep learning framework to extract crucial feature maps from three distinct datasets. Classification was carried out using an optimized ResNext model, leveraging the Wildebeest Herd Optimization (WHO) algorithm. To assess the model’s performance, the Leukocyte Images for Segmentation and Classification (LISC) dataset samples were utilized as input, resulting in an impressive accuracy of 99.16% for these images.

CNN-based architectures have been extensively employed in the majority of studies published in the literature. However, CNNs heavily rely on local receptive fields and pooling operations, which impose limitations on their capability to capture long-range dependencies within an image. This constraint impedes their potential to acquire a comprehensive understanding of the input and comprehend intricate relationships among various regions of the image. Additionally, CNN architectures frequently necessitate meticulous engineering and optimization efforts to attain optimal performance on particular datasets, thereby reducing their flexibility and adaptability. Unlike traditional CNNs that use spatial convolutions to extract features from images, Vision Transformer (ViT) models that use self-attentional mechanisms to capture the relationships between different regions of an image can improve performance [22]. ViT models have significant advantages over traditionally used deep learning architectures. Firstly, ViT models offer a more general and universal architecture. To process visual data, these models first decompose the image into small patches and then process these patches with a set of attention mechanisms [23]. This approach allows the model to learn more general features and detect objects at different scales. The application of transformer-based approaches for classifying medical images is an emerging field of research [24]. Presently, numerous studies focusing on disease detection using transformer-based models have been presented. Wu et al. [25] proposed a ViT model for the classification of emphysema subtypes using CT images. The dataset samples underwent preprocessing, followed by training of five distinct CNN-based models (AlexNet, Inception-V3, MobileNet-V2, ResNet34, and ResNet50) alongside the ViT model. he ViT model exhibited superior performance in comparison to the CNN-based models, achieving an accuracy rate of 95.95%. Feng et al. [26] developed the ViT-Patch model for the detection of benign and malignant tumors in ultrasound images. Ammar et al. [27] proposed the ViT-TB model specifically designed for the identification of Tuberculosis disease from X-Ray images.

Although ViT models have shown very high performance in studies on medical images, they are not reliable for clinical use. The main reason for this is that the areas they focus on in their predictions are not known. This black box structure, which is also valid for CNN-based models, can be overcome with various CAM algorithms. Oztekin et al. [28] proposed a CNN-based explainable model for detecting caries in panoramic dental images. The model visualizes the pixel areas it focused on in its predictions using the Grad-CAM algorithm. Lee et al. [29] developed a pre-trained CNN-based model for identifying acute intracranial hemorrhage. The model visualizes the specific areas it focuses on in its predictions through the utilization of the Grad-CAM. CAM algorithms, commonly employed to provide interpretability to CNN-based models, utilize the outputs of the final convolutional layer preceding the classifier layer. However, since ViT models lack any convolutional layer prior to the classifier, the outputs required for feeding CAM algorithms become intricate. Consequently, the application of CAM algorithms with ViT models remains limited in the number of studies conducted.

This paper proposes an explainable ViT model for computer-assisted automatic WBCs classification. The proposed model first divides the input image into 16 × 16 pixel patches and then processes these patches in encoder blocks to predict the class. The model visualizes the specific pixel areas it emphasizes in its predictions utilizing the Score-CAM algorithm. The approach proposed in this study has the potential to be applied clinically for computer-aided automatic WBC detection due to its explainable structure. Moreover, to the best of the author’s knowledge, this is the first study on Raabin-WBC dataset classification from blood films using explainable ViT model.

The main contributions of this study can be summarized as follows:

- Using Raabin-WBC, a dataset that contains five different types of WBCs and is more comprehensive than previous datasets, classification is performed with the ViT deep learning model.
- A method is proposed to visualize the areas that the ViT model focuses on in its predictions with the Score-CAM algorithm.
- In the predictions made on the input images, which are divided into 16 × 16 pixel patches and then vectorized, the class probabilities are calculated with the softmax.
- The proposed method shows high accuracy and precise localization. The model also achieves high softmax values in successful predictions, outperforming CNN models in similar studies.

## 2. Materials and Methods

**Figure 1:**
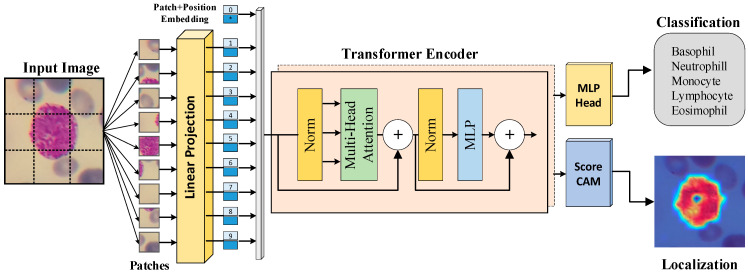
Block representation of the proposed WBC classification and localization method.

This study presents a cutting-edge deep learning model for the accurate identification and classification of various subtypes of WBCs using the ViT model. This approach utilizes a dataset of images of WBCs to train the model, enabling it to accurately classify the cells into different subtypes with high accuracy. The model takes an image of a WBC as input and employs its deep learning capabilities to output a prediction of the WBC’s subtype. In this prediction process, the Score-CAM algorithm is utilized to demonstrate which regions within the image influence the classification decision. The proposed approach is illustrated in the diagram in Figure 1.

### 2.1. WBCs Dataset

**Figure 2:**
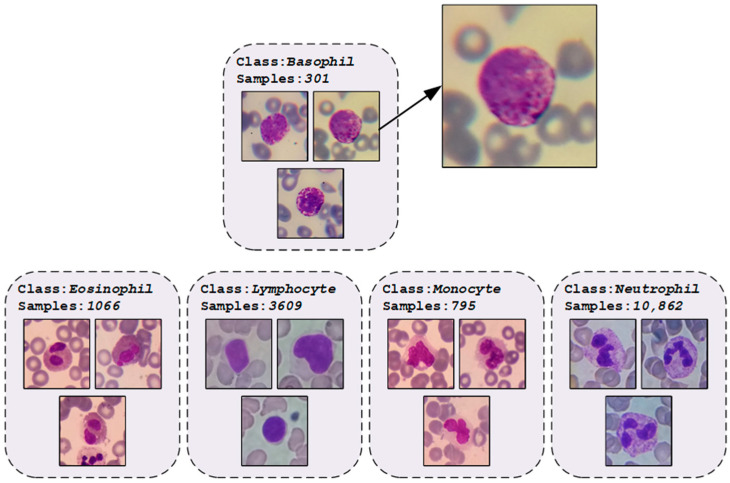
An illustration of the dataset with number of classes and some class images.

In this study, we utilized the publicly available dataset Raabin-WBC [30], created from 72 regular peripheral blood films. The samples in the dataset were stained using the Giemsa technique and viewed at 100× magnification using two microscopes. Additionally, smartphones equipped with an adapter designed and fabricated via 3D printing were employed to capture images by mounting the phone to the microscope ocular lens. A total of approximately 23,000 images were acquired and processed utilizing a color filter and a Faster RCNN network to extract WBCs. The data were further cleaned to eliminate duplicate cell images and a comprehensive labeling process was undertaken to accurately determine the cell types. The classes in the dataset obtained after the labeling process and the number of samples they contain are presented in Figure 2.

Upon examination of the class distributions of the Raabin-WBC dataset samples, it becomes apparent that the dataset is unbalanced. While this may initially be perceived as a problem for use in model training, it is in fact more suitable for real-world data. This is because the distribution of WBCs in the population is not equal, with certain types of WBCs being more prevalent than others. An unbalanced dataset accurately reflects this reality and allows more accurate diagnosis and treatment. Conversely, a balanced dataset may lead to oversampling of less common WBCs, resulting in skewed results and inaccurate conclusions. Additionally, a balanced dataset may lead to oversampling of certain types of WBCs, thereby biasing the training of a machine learning model and resulting in poor performance on real-world data. For this reason, no data augmentation method was applied to the dataset samples. A total of 80% of the dataset samples were reserved for training, 10% for validation, and 10% for testing. The number of data used at each stage as a result of the splitting process is presented in Table 1.

### 2.2. Transformer-Based Image Classification Method

**Figure 3:**
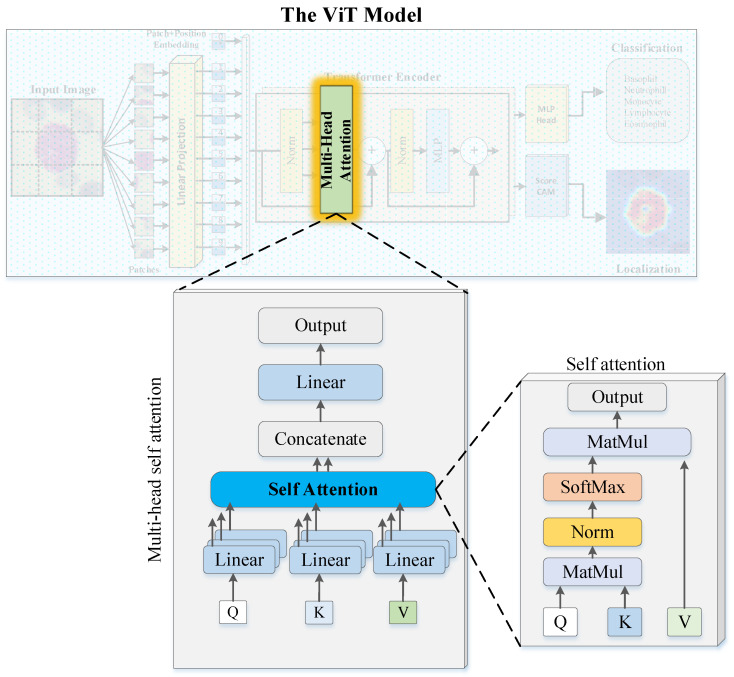
The block diagram depicts the multi-head self-attention (MHA) mechanism.

Transformers are a type of neural network architecture that have been widely utilized in natural language processing (NLP) tasks, such as language translation and language modeling [31]. The transformer architecture is based on the concept of self-attention, which enables the model to weigh the importance of different parts of the input when making predictions. Self-attention is implemented through an attention mechanism, which calculates a weighted sum of the input values based on their relationships to a particular position or query [32]. This mechanism allows the transformer to learn relationships between different parts of the input, which is particularly beneficial in NLP tasks where the meaning of a word or phrase depends on its context. Furthermore, the transformer architecture also employs an encoder–decoder structure [33]. The encoder takes in the input and generates a set of hidden representations, which are then passed to the decoder to generate the output. Both the encoder and decoder are composed of multiple layers, each containing multiple self-attention mechanisms. This enables the model to learn and extract information from the input at multiple levels of abstraction, which is essential for understanding the meaning of the input. The block diagram depicting the multi-head self-attention (MHA) mechanism employed in this study is presented in Figure 3.

In the context of MHA, each patch of the input vector undergoes a self-attention process, wherein it is transformed into three distinct vectors: query (Q), key (K), and value (V), through the use of weight matrices [34]. The saliency of each patch is determined by calculating the dot product of its query and key vectors, producing a score matrix. The softmax activation function is then applied to the score matrix, generating attention weights. Finally, the attention weights are multiplied with the value vector, resulting in the self-attention output. After the self-attention mechanism to each patch and the computation of resulting self-attention matrices, they are aggregated and processed by a linear layer. Subsequently, a regression head is employed to generate the final output of the MHA mechanism.

Dosovitskiy et al. [35] proposed the ViT model, which is based on transformers and comprises self-attention blocks and multilayer perceptron (MLP) networks. ViTs are similar to standard transformers, but are designed to handle images as input. Specifically, ViTs divide images into smaller non-overlapping patches and then use a transformer architecture to process each patch separately [36]. This enables the model to learn and extract information from the images at multiple levels of abstraction, similar to how the transformer architecture works for NLP tasks. A key feature of ViTs is their ability to handle images as input without the need for a pre-processing step, such as applying convolutional layers. Instead, ViT divide images into smaller non-overlapping patches and then use a transformer architecture to process each patch separately. A particularly noteworthy aspect of ViT is their use of a linear projection to reduce the dimensionality of image patches before feeding them into the transformer network. This approach may seem counterintuitive as it reduces the information in the image patches. However, this linear projection serves a crucial role in the ViT architecture and has several benefits. It allows for a more computationally efficient model. By reducing the dimensionality of the image patches, the model can be trained on larger images without requiring a large amount of computational resources, making ViT more accessible to researchers and practitioners and allowing for more widespread usage and experimentation.

The base model of the ViT architecture is used in this study. The details of the model consisting of 12 Encoder blocks are presented in Table 2 (model available at: github.com/oguzhankatar/ViT/). A ViT model with input size (1, 3, 224, 224) accepts an input image with 3 channels (RGB) and 224 × 224 pixels. Then, the Conv2D (Projection) layer starts by splitting the input image into a series of small pieces. These fragments are typically called “patches” and each one represents a part of the image. The Conv2D layer subtracts the patches using a 16 × 16 filter. ViT performs a transform for each patch using the Conv2D layer. These transformations convert the image fragments into a set of vector representations, and these vectors are processed in the encoder blocks of the ViT model.

Each Encoder Block receives an input of the form (1, 197, 768) and produces an output of the same form (1, 197, 768). Encoder blocks consist of an attention mechanism, an intermediate layer, and an output layer. The attention mechanism is used in the ViT model to extract features of the image and highlight important information. The intermediate layer is used to transform the output of the attention mechanism into more complex and rich features. This layer consists of the Dense layer and an activation function called GELUActivation. The output layer resizes the output of the intermediate layer back to the original dimensions and includes a Dropout layer. Before the input and after the output of each encoder block are LayerNorm layers. LayerNorm normalizes the output and applies weighted normalization operations. This helps the model to run stably and robustly. The Normalization layer after Encoder Block-12 normalizes the data of size (1, 768) and produces an output of size (1, 5). Then, a Linear layer is used to transform the data into class labels of (1, 5). In this way, predictions can be made for five different classes of WBCs.

## 3. Experiments

### 3.1. Experimental Setup

In this study, a pre-trained ‘vit-base-patch16-224’ model with the ImageNet-21k (14 million images, 21,843 classes) dataset was utilized due to the high cost of training a ViT model from scratch with random weights. Timm and PyTorch libraries were used in the implementation of the model. The output layer of the model was modified to match the number of classes in the dataset. In the pre-processing stage, only the input images were resized according to the ViT model. As the default input size of the pre-trained ViT model is 224 × 224, all of the dataset samples were resized to this size. The model was then trained on the samples allocated for the training set using the AdamW optimizer and the CrossEntropyLoss function. During training, the pre-trained weights of the model were constantly updated to better fit the set of WBCs. The batch size value was kept constant at 16, and the learning rate at 0.00002. The maximum number of epochs was set to 100, but an early stopping function was defined to monitor the validation loss value. If there was no decrease in the validation loss for five consecutive epochs, the early stopping function would terminate the model training, and the weights of the epoch with the highest classification ability would be recorded. A brute-force approach was used to determine these parameters. The parameters that give the most optimum result were determined by studies on the validation set. Once the parameters were established, all experiments were conducted in the Google Colab environment.

### 3.2. Performance Evaluation Metrics

**Figure 4:**
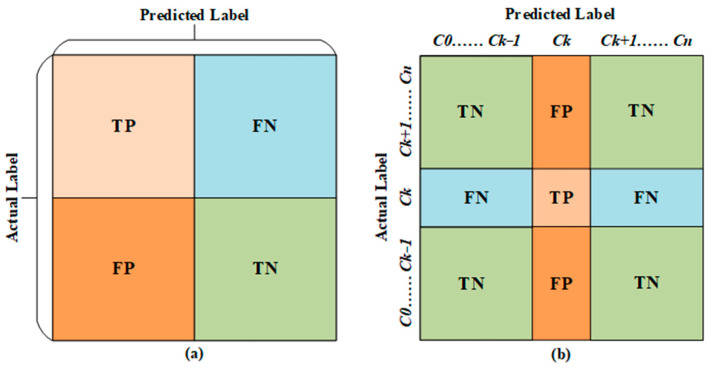
Confusion matrices for (**a**) binary-class and (**b**) multi-class scenarios.

In the evaluation of deep learning models for classification tasks, confusion matrix-based metrics are commonly employed. A confusion matrix illustrates the correspondence between the model’s predicted class label for a given input image and the true class label of that image. In artificial intelligence-based classification studies, various situations may arise in the output layer of the model. However, these situations can be encapsulated by four metrics: True Positive (TP), True Negative (TN), False Positive (FP), and False Negative (FN). Figure 4 illustrates the placement of these metrics in the fields of multi-class and binary classification studies, with respect to a random class.

Evaluation of an image classifier deep learning model can be accomplished utilizing a variety of performance metrics, including accuracy, precision, recall, and F-1 score. Accuracy, which is the proportion of correct predictions made by the model, is calculated as the number of correct predictions divided by the total number of predictions. Recall, which is a measure of the model’s ability to detect all positive instances, is calculated as the number of true positive predictions divided by the total number of actual positive instances. Precision is a measure of the model’s ability to correctly identify positive instances, and is determined by dividing the number of true positive predictions by the total number of positive predictions. The F-1 score, which is the harmonic mean of precision and recall, is a useful metric for balancing precision and recall when they are in conflict. The mathematical definitions for these measures are provided below.

Accuracy (Acc) = (TP + TN)/(TP + FP + FN + TN)(1)

Recall (Rec) = TP/(TP + FN)(2)

Precision (Pre) = TP/(TP + FP)(3)

F-1 Score (F1) = (2 × (Pre × Rec))/(Pre + Rec)(4)

### 3.3. Results

**Figure 5:**
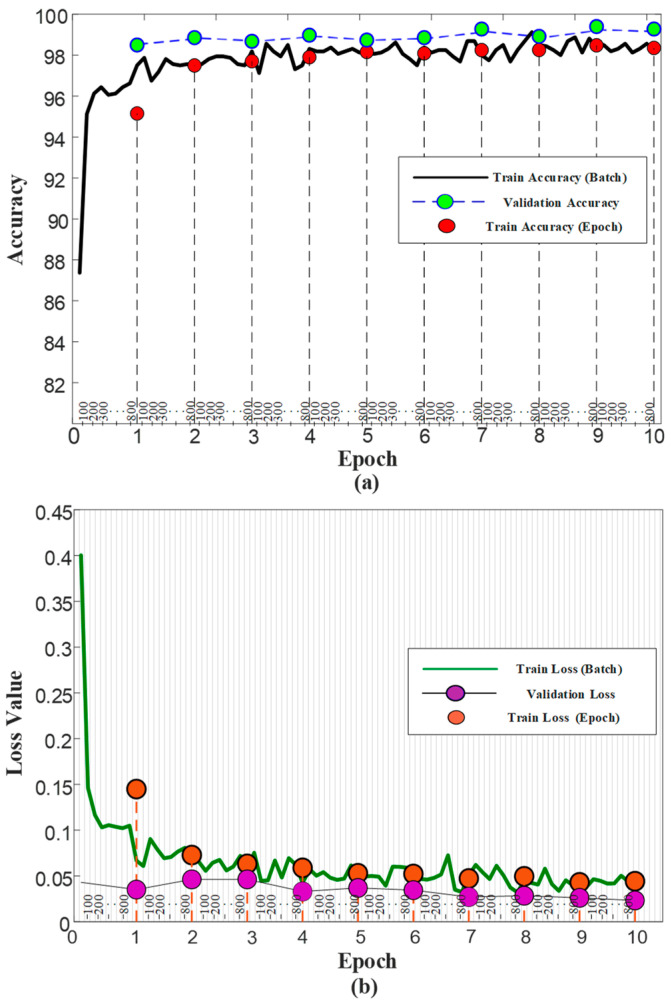
The ViT model performance curves during training. (**a**) Accuracy values and (**b**) Loss values.

Using a pre-trained model can have several implications for the training phase. The pre-trained model provides a starting point that is already optimized, reducing training time and saving computational resources compared to training a model from scratch. Pre-trained models often perform better than models trained from scratch as they have already learned general representations from a large dataset, providing a strong foundation for fine-tuning the target task. Utilizing the pre-trained models, the training duration for the ViT model was set to a maximum of 100 epochs. However, the activation of the early stopping function resulted in the cessation of training at the 10th epoch, and the weights were saved in the ‘.pth’ format. During the training process, each epoch was completed in an average of 47 s. The accuracy and loss graphs generated during the training phase of the model are illustrated in Figure 5.

**Figure 6:**
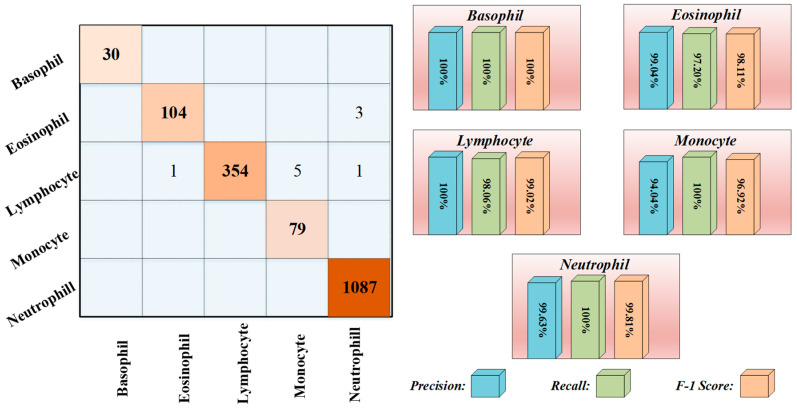
The confusion matrix and some graphs of metrics for multi-class for test dataset.

The performance of the proposed ViT model on both the training and validation sets was consistent with the expectations based on the pre-trained models and reached the desired level. Specifically, the model attained a validation accuracy of 99.27% and a validation loss of 0.02% at the end of the 10th epoch. However, test images were utilized to assess the model’s capability to generalize to new and unseen scenarios, which is crucial in practical applications. The confusion matrix and recall, precision, and F-1 score graphs obtained from the testing phase are presented in Figure 6. The ViT model achieved an accuracy of 99.40%, with only 10 misclassifications on a set of 1664 test images.

**Figure 7:**
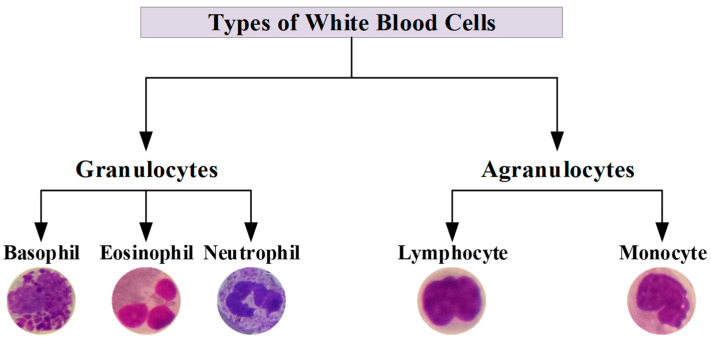
A schematic representation for types of white blood cells.

Upon examination of the test results, it was observed that exceptional performance scores were obtained for each class. However, the precision value for the Monocyte class was lower than the other classes. The primary cause for this is the overestimation of samples as Monocytes, despite their actual classification as Lymphocytes. These two classes of samples share similar visual features and do not contain granules. WBCs are commonly classified into five distinct types. However, based on their morphological characteristics, it is feasible to divide the cells into two groups: Granulocytes and Agranulocytes. The cells that constitute Granulocytes and Agranulocytes are depicted in Figure 7.

**Figure 8:**
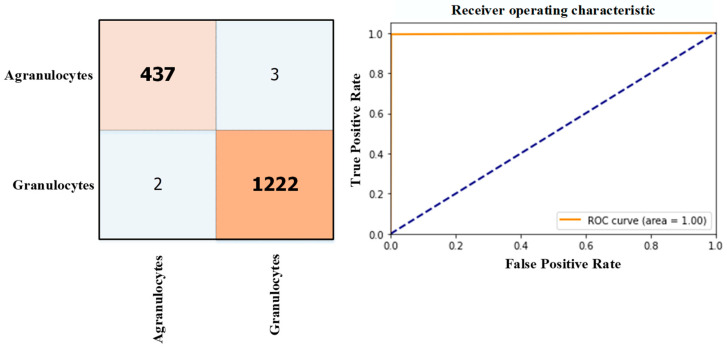
The ViT model performance on Granulocytes and Agranulocytes classification.

Samples of datasets for binary classification of WBCs are annotated under the classification scheme depicted in Figure 7. The datasets were trained using the same data split ratios and hyperparameters as in the 5-class classification task. The ViT model demonstrated the capability to differentiate between Granulocytes and Agranulocytes, achieving an accuracy of 99.70% on the test samples. The results of the testing phase, including the confusion matrix and ROC curve, are presented in Figure 8.

**Figure 9:**
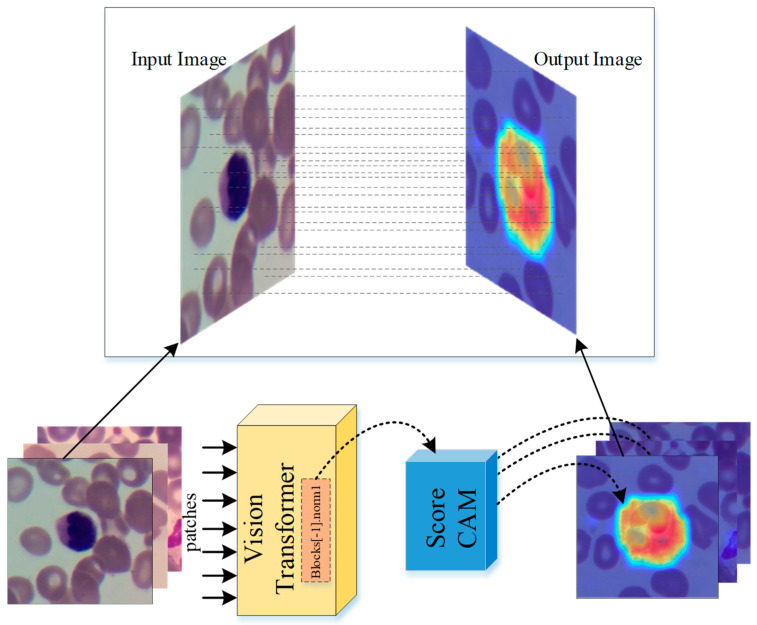
The operational framework of the explainable ViT model.

The traditional view of deep learning models has been that they are black boxes due to their complex structure, making it challenging to comprehend the internal workings of the models and how they arrive at a specific decision. However, recent advancements in explainability techniques have altered this perception, enabling us to shed light on the inner workings of deep learning models [37,38]. One such technique is Score-CAM [39], which allows visualization of the most important features of the input data for the model’s decision-making process. With the application of these techniques, deep learning models have transformed from being black boxes to becoming more explainable. This is crucial in applications where the model’s decisions have significant consequences, as it enables the building of trust and confidence in the model. The aggregate performance measures, such as accuracy, recall, and precision, only provide a general view of the model’s performance and do not reveal the underlying mechanisms that drive the model’s decisions. On the other hand, Score-CAM-like algorithms offer a way to understand the model’s behavior and decision-making process, which is vital for establishing accountability and trust in the model. In this study, the predictions made by the ViT model were explained by utilizing the Score-CAM algorithm, which was used to focus on and highlight the areas of interest. ViT models are characterized by the utilization of self-attention mechanisms, which enable the model to focus on relevant parts of the input image while disregarding irrelevant ones. One of the notable characteristics of ViT models is the output of their layers, which is typically of the shape BATCH × 197 × 192. In this dimension, the first element represents the class token, while the remaining 196 elements represent the 14 × 14 patches in the image. The class token is employed in making the final classification, and the remaining 196 elements can be viewed as a 14 × 14 spatial image with 192 channels. To integrate the Score-CAM algorithm into vision transformers, it is necessary to reshape them into 2D spatial images. This can be accomplished by passing a reshape transform function to the CAM constructor. By doing so, it is possible to visualize the regions of the input image that are most crucial for the model’s final classification. The final classification is based on the class token computed in the last attention block. As a result, the output of the model is not affected by the 14 × 14 channels in the last layer, and the gradient of the output with respect to them is 0. Therefore, it is recommended to choose any layer prior to the final attention block when generating CAMs to better understand the model’s behavior. The operational framework of the explainable ViT model proposed in this study, along with its constituent layers, is depicted in Figure 9.

**Figure 10:**
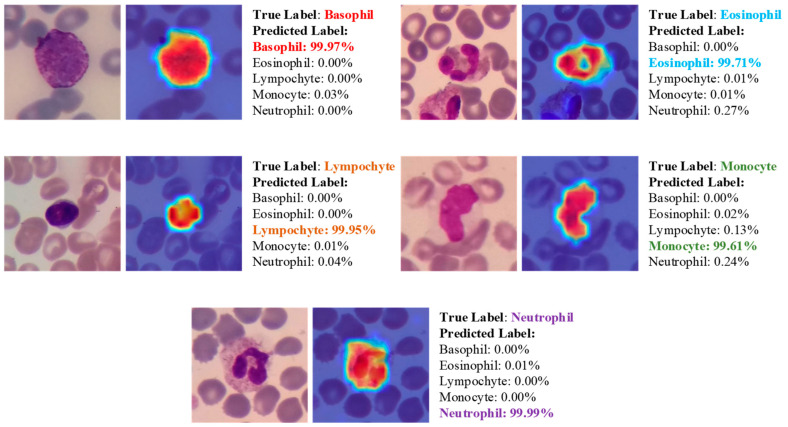
The areas upon which the model correctly focuses its predictions on the test images.

The areas upon which the model correctly focuses its predictions on the test images are presented in Figure 10. The regions of focus identified by the ViT model exhibit a significant overlap with the areas of WBCs.

**Figure 11:**
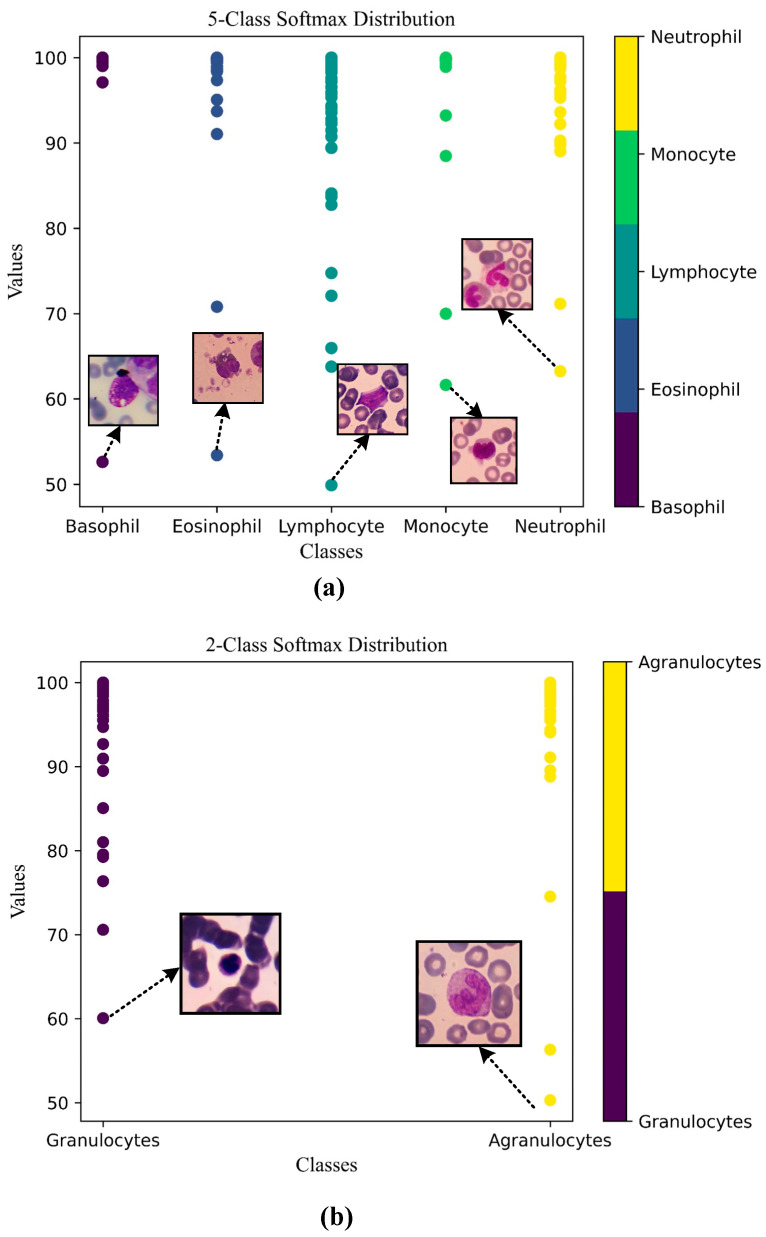
Class softmax distribution on true predicted samples (**a**) 5-Class softmax distribution and (**b**) 2-Class softmax distribution.

This alignment between the Score-CAM output and the ground truth is a promising indication that the model is effectively learning meaningful features from the input data and utilizing these features to make accurate predictions. The softmax probability percentages exhibited by the model in its true predictions on the test images are given in Figure 11.

Performing the 5-class classification task, the model produced an average softmax probability of 98.25% for Basophil class samples, 98.92% for Eosinophil class samples, 98.97% for Lymphocyte class samples, 98.82% for Monocyte class samples and 99.84% for Neutrophil class samples. The prediction with the lowest probability was 49.89% for a sample from the Lymphocyte class.

Performing the 2-class classification task, the model produced an average softmax probability of 99.77% for Granulocytes class samples, 99.42% for Agranulocytes class samples. The prediction with the lowest probability was 50.30% for a sample from the Agranulocytes class.

**Figure 12:**
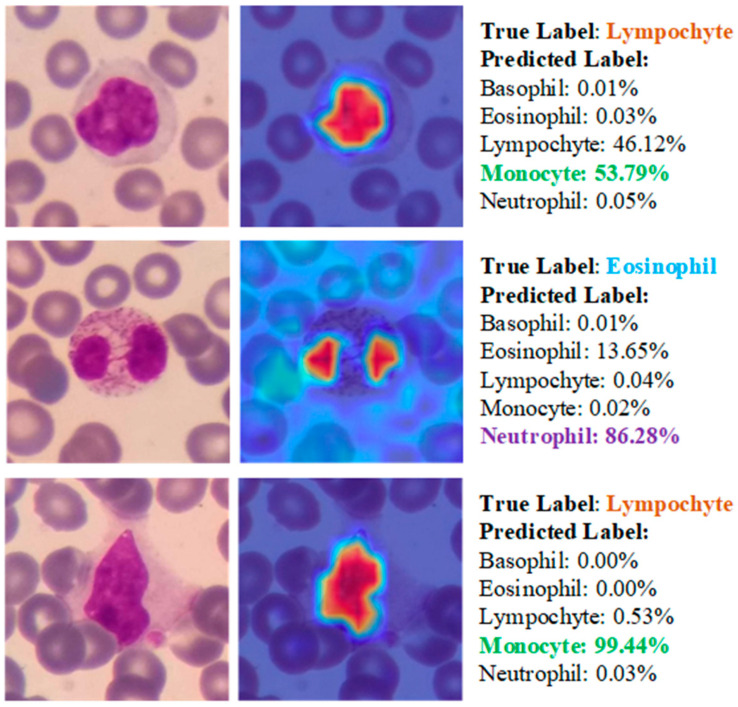
Some examples of misclassified samples and class probabilities during test phase.

Besides accurate predictions, analysis of Score-CAM outputs for images misclassified by the ViT model can provide valuable information about the strengths and weaknesses of the model. Figure 12 illustrates a few examples of misclassified images and probabilities.

In the case of incorrectly predicted images, the model continued to focus on WBCs areas. However, the inaccuracies in the model’s predictions could be attributed to visual similarities between cells.

## 4. Discussion

WBCs classification plays a crucial role in diagnosing of many diseases, including infections and blood-related disorders. Although WBC classification is a process that can be easily performed in a laboratory environment, the automation of basic problems by machine learning is valuable in the field of health as in every field. The achievements in the field of deep learning, which started with CNN, are developing with different architectures every day. This study covers an application of recently popularized ViTs in WBC detection. Table 3 provides details for a hand-curated selection of research studies on that topic. Tavakoli et al. [40] introduced a novel approach for the classification of white blood cells utilizing image processing and machine learning techniques. The proposed method encompasses three main stages, namely nucleus and cytoplasm detection, feature extraction, and classification through an SVM model. The achieved accuracy rate of the proposed method in categorizing WBCs in the Raabin-WBC dataset was 94.65%. Katar and Kilincer [41] proposed an approach for the automatic classification of WBCs using pre-trained deep learning models, including ResNet-50, VGG-19, and MobileNet-V3-Small. The proposed approach achieved high accuracy rates, with the MobileNet-V3-Small model reaching the highest accuracy of 98.86%. Akalin and Yumusak [42] present a study on the real-time detection and classification of WBCs in peripheral blood smears using object recognition frameworks. YOLOv5s, YOLOv5x, and Detectron2 R50-FPN pre-trained models were used, and two original contributions were made to improve the model’s performance. The maximum accuracy rate achieved on the test dataset for detection and classification of WBCs was 98%. Leng et al. [43] present a study on developing a deep learning-based object detection network for leukocyte detection using the detection transformer (DETR) model. The study findings indicate that the improved DETR model outperforms the original DETR and CNN with a mean average precision (mAP) detection performance of up to 96.10%.

In this study, the pre-trained ViT model was utilized for automatic classification of white blood cells. The model trained for five distinct types of white blood cells attained an accuracy rate of 99.70%. In contrast, the fine-tuned model, which classified cells based on their granule content, achieved an accuracy rate of 99.40%. In comparison to the studies listed in Table 3, we achieved higher accuracy and evolved the ViT model into an explainable structure using the Score-CAM algorithm. The ViT model’s superior performance can be attributed to its unique architecture, which enables it to capture long-range dependencies between different parts of the image, resulting in better image recognition performance.

The advantages of our explainable model can be summarized as follows:

- The proposed model is based on vision transformers that has become popular research field. Therefore, this study is an example to examine vision transformers performance in biomedical image classification.
- Since the ViT model used in the study was trained on large datasets, it performed well on the WBC classification problem with a low training cost.
- This model can classify WBCs images with end-to-end transformer structure. There is no need to use any feature engineering.
- Due to the explainable structure, the proposed model presents focused regions during the classification process. According to these results, experts can validate model performance.
- Due to its high level of classification accuracy, it has the potential to be utilized in clinical applications.
- Trained with the Rabbin-WBC dataset, the model can be fine-tuned to classify different cell types and can be easily implemented.

The limitations of our study are outlined as follows. Although the proposed method achieves a high success rate in classifying WBCs, its response time was not assessed in a real-time study. Additionally, the model’s resilience to image variations due to factors such as illumination and the noise was not verified. To address these limitations, future research will involve generating synthetic images using data augmentation techniques and training new models with these images. The computational and memory requirements of these models are higher than other models. Processing large-sized images increases the computational intensity and requires more memory space. Future work will be performed to optimize the complex parameters of the network. The dataset used in the study is a public WBC data. In future studies, machine learning studies for different cell-based problems will be carried out in collaboration with clinical pathologists.

## 5. Conclusions

In this study, we propose an explainable method based on the vision transformer for the automatic detection of white blood cells in blood film images. The model is trained and validated using a public five-class dataset of 16,633 samples. The pre-trained ViT model achieved a testing phase accuracy rate of 99.40% for the detection of five different subtypes of white blood cells. Examination of the model’s predictions revealed that the most misclassified samples belonged to the Lympochyte subtype, which was predominantly predicted as Monocyte. Since Lympochyte and Monocyte cells lack granules and share similar visual features, this misclassification is understandable. The dataset used to confirm this situation was labeled according to granule presence, and the ViT model was trained using binary classification. The resulting model correctly classified test images with a success rate of 99.70%. The pixel areas focused on in the ViT model’s predictions were visualized using a heat mapping technique with the Score-CAM algorithm, further enhancing the model’s reliability. The study’s main limitation is the lack of information on real-time performance compared to object detection algorithms. The proposed ViT model can automate the detection of cells in blood films and can be effectively used in medical education due to its explainable structure. Moreover, the model can be fine-tuned for similar tasks, benefiting from the knowledge accumulated during training and achieving high accuracy rates.

## Figures and Tables

**Table 1:** Data distribution at each stage of the splitting process for training, validation, and testing.

Phase	Basophil	Eosinophil	Lymphocyte	Monocyte	Neutrophil	Total
Training	241	852	2887	637	8688	13,305
Validation	30	107	361	79	1087	1664
Testing	30	107	361	79	1087	1664
Total	301	1066	3609	795	10,862	16,633

**Table 2:** Proposed ViT model architecture.

Layer Name	Input Shape	Output Shape
PatchEmbed	(1, 3, 224, 224)	(1, 196, 768)
Conv2D (proj)	(1, 3, 224, 224)	(1, 768, 14, 14)
Idenity (norm)	(1, 196, 768)	(1, 196, 768)
Dropout	(1, 197, 768)	(1, 197, 768)
Idenity (patch_drop)	(1, 197, 768)	(1, 197, 768)
Idenity (norm_pre)	(1, 197, 768)	(1, 197, 768)
Encoder Block-1	(1, 197, 768)	(1, 197, 768)
LayerNorm (norm1)	(1, 197, 768)	(1, 197, 768)
Attention	(1, 197, 768)	(1, 197, 768)
Idenity (ls1)	(1, 197, 768)	(1, 197, 768)
Idenity (drop_path1)	(1, 197, 768)	(1, 197, 768)
LayerNorm (norm2)	(1, 197, 768)	(1, 197, 768)
MLP	(1, 197, 768)	(1, 197, 768)
Idenity (ls2)	(1, 197, 768)	(1, 197, 768)
Idenity (drop_path2)	(1, 197, 768)	(1, 197, 768)
Encoder Block-2	(1, 197, 768)	(1, 197, 768)
Encoder Block-3	(1, 197, 768)	(1, 197, 768)
Encoder Block-4	(1, 197, 768)	(1, 197, 768)
Encoder Block-5	(1, 197, 768)	(1, 197, 768)
Encoder Block-6	(1, 197, 768)	(1, 197, 768)
Encoder Block-7	(1, 197, 768)	(1, 197, 768)
Encoder Block-8	(1, 197, 768)	(1, 197, 768)
Encoder Block-9	(1, 197, 768)	(1, 197, 768)
Encoder Block-10	(1, 197, 768)	(1, 197, 768)
Encoder Block-11	(1, 197, 768)	(1, 197, 768)
Encoder Block-12	(1, 197, 768)	(1, 197, 768)
LayerNorm (norm)	(1, 197, 768)	(1, 197, 768)
Idenity (fc_norm)	(1, 768)	(1, 768)
Dropout	(1, 768)	(1, 768)
Linear (head)	(1, 768)	(1, 5)

**Table 3:** Comparison of our work with some state-of-the-art study techniques for WBC classification.

Study	Year	Number of Class	Method	Explainability	Performance
Tavakoli et al. [40]	2021	5 (Basophil, Eosinophil, Lymphocyte, Monocyte, Neutrophil)	SVM	Black-box	Acc = 94.65%
Katar and Kilincer [41]	2022	5 (Basophil, Eosinophil, Lymphocyte, Monocyte, Neutrophil)	CNN	Grad-CAM	Acc = 98.86%
Akalin and Yumusak [42]	2022	5 (Basophil, Eosinophil, Lymphocyte, Monocyte, Neutrophil)	Hybrid	Black-box	Acc = 98.00%
Leng et al. [42]	2023	3 (Eosinophil, Monocyte, Neutrophil)	DETR	Black-box	mAP = 96.10%
The proposed study	2023	2 (Granulocytes and Agranulocytes)	ViT	Score-CAM	Acc = 99.70%
		5 (Basophil, Eosinophil, Lymphocyte, Monocyte, Neutrophil)			Acc = 99.40%

## Data Availability

Not applicable.
